# Conditional Random Fields and Supervised Learning in Automated Skin Lesion Diagnosis

**DOI:** 10.1155/2011/846312

**Published:** 2011-10-20

**Authors:** Paul Wighton, Tim K. Lee, Greg Mori, Harvey Lui, David I. McLean, M. Stella Atkins

**Affiliations:** ^1^Department of Computing Science, Simon Fraser University, Burnaby, BC, Canada V5A 1S6; ^2^Department of Dermatology and Skin Science, Photomedicine Institute, University of British Columbia and Vancouver Coastal Health Research Institute, Vancouver, BC, Canada V5Z 4E8; ^3^Cancer Control Research Program and Integrative Oncology Department, BC Cancer Research Centre, Vancouver, BC, Canada V5Z 4E6

## Abstract

Many subproblems in automated skin lesion diagnosis (ASLD) can be unified under a single generalization of assigning a label, from an predefined set, to each pixel in an image. We first formalize this generalization and then present two probabilistic models capable of solving it. The first model is based on independent pixel labeling using maximum a-posteriori (MAP) estimation. The second model is based on conditional random fields (CRFs), where dependencies between pixels are defined using a graph structure. Furthermore, we demonstrate how supervised learning and an appropriate training set can be used to automatically determine all model parameters. We evaluate both models' ability to segment a challenging dataset consisting of 116 images and compare our results to 5 previously published methods.

## 1. Introduction

Incidence rates of melanoma are increasing rapidly in the western world, growing faster than any other cancer [1]. Since there is no effective therapy for patients with advanced melanoma [2], educational campaigns attempt to encourage high-risk individuals to undergo routine screening so that melanomas can be identified early while they are still easily treatable [3]. While worthwhile, these educational campaigns generate a large amount of referrals to dermatologists, whose services are already undersupplied [4].

**Figure 1:**
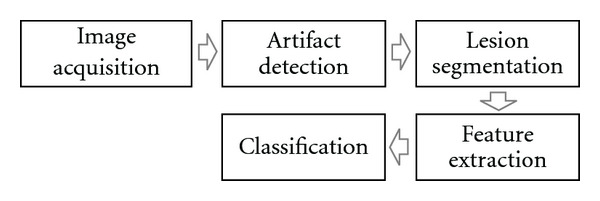
Typical computer aided diagnosis (CAD) pipeline usually adopted for automated skin lesion diagnosis (ASLD). Our goal is to (1) generalize the artifact detection, segmentation as well as a portion of the feature extraction stage into a single mathematical framework and (2) propose and evaluate probabilistic models which employ supervised learning to quickly and automatically “learn” to perform these tasks.

Automated skin lesion diagnosis (ASLD) is expected to alleviate some of this burden. By acting as a screening tool, ASLD can reject obviously benign lesions, while referring more suspicious ones to an expert for further scrutiny. Most ASLD methods adopt the standard computer-aided diagnosis (CAD) pipeline illustrated in Figure 1. First an image is acquired with a digital dermoscope. Next, undesirable artifacts (such as hair or oil bubbles) are identified and, if necessary, replaced with an estimate of the underlying skin color. After this, the lesion is segmented, and discriminative features are then extracted. Finally, supervised learning is used to classify previously unseen images.

Our previous work demonstrated how the use of supervised learning, under the proper generalization (of assigning labels to pixels), was able to solve several tasks in this pipeline including detecting occluding hair, segmenting the lesions, and detecting the dermoscopic structure *pigment network* [5]. Our method was relatively simple; it labeled pixels in an image independently using modest features, linear discriminant analysis (LDA) for supervised dimensionality reduction, and maximum a-posteriori (MAP) estimation. Nevertheless, in spite of its simplicity, our model was able to perform comparably to other previously published, nongeneral methods for lesion segmentation [6–10] and hair detection [11].

In this paper, we seek to expand on this generalization by replacing the per-pixel (PP) estimation model with a conditional random field (CRF) model. The largest criticism levied at the PP approach is that pixels are labeled independently, regardless of the label assigned to their neighbors. This assumption of independence is clearly not valid, as there is a high degree of correlation between neighboring pixels in any image (any image other than pure noise, i.e.). CRF-based models attempt to relax this assumption of independence by creating a graphical model which defines the dependencies between pixels.

In order to apply a CRF model, a parameter vector specifying the relative contribution of the input features is required. Often, these parameters are determined in an ad hoc fashion via trial and error. Since our goal is a *general* method, easily applicable to a variety of problems, it is crucial that these parameters be determined automatically based on observations. We, therefore, apply the maximum likelihood estimator for the parameter vector and describe a gradient-based method for its computation. We also address many practical considerations encountered during the implementation.

The paper is organized as follows: in Section 2, we briefly review previous work. In Section 3, we formulate the generalization in Section 3.1, review our previous PP model [5] in Section 3.2, and present our CRF model in Section 3.3. In Section 4, we present results. Finally, we conclude in Section 5.

## 2. Previous Work

Our original PP model was based on the work by Debeir et al. [12] who also attempts to generalize many tasks in ASLD. Our model was found to perform comparably to many published lesion segmentation algorithms including K-means++ (KPP) [6], J-image segmentation (JSEG) [7], dermatologist-like tumor area extraction algorithm (DTEA) [8], statistical region merging (SRM) [9], and threshold fusion (FSN) [10]. It also performed comparably to DullRazor [11] for detecting occluding hair and was able to identify the dermoscopic structure *pigment network*. Our PP model is briefly reviewed in Section 3.2; however, we refer readers to our previous study for further details, as well as a more comprehensive review of previous work in ASLD, including the methods against which we compare [5].

We defer the review of the CRF model until Section 3.3, where we examine it in detail.

## 3. Method

This section is divided into 3 parts. We begin in Section 3.1 by formalizing the generalization that is capable of performing a variety of tasks in ASLD. In Section 3.2, we briefly review our previous PP model [5]. Finally, in Section 3.3, we outline our CRF model.

### 3.1. The Generalization

We are given a set of observations {*x*^*m*^, *y*^*m*^}, consisting of images (*x*) and corresponding ground truths labeling (*y*). Using the notation of Szummer et al. [13], the superscript *x*^*m*^ or *y*^*m*^ indexes a specific image/labeling in the set and the subscript *x*_*i*_ or *y*_*i*_ indexes a specific pixel. Let *N*_*I*_ represent the number of images, and *N*_*P*_^*m*^ represents the number of pixels in image *x*^*m*^. An imageset can contain any number of channels (or features), which we denote by *N*_*C*_. Valid values for each entry in the label field (*y*_*i*_) are defined by the label set *L* = {*l*_1_,…, *l*_*N*_*L*__}, where *N*_*L*_ is the number of possible labels.

Given our training set {*x*^*m*^, *y*^*m*^}, we use supervised learning to predict the label fields for previously unseen images.

Formally, we are given

(1) $$\begin{array}{c} \left\{x^{m},y^{m}\right\};\;m=1,\ldots ,N_{I};\;i.i.d,\\ L=\left\{l_{i}\right\};\;i=1,\ldots ,N_{L};\;l_{i}\in \mathbb{N},\\ x^{m}\in {\mathbb{R}}^{N_{P}^{m}\times N_{c}},\\ y^{m}\in L^{N_{P}^{m}}. \end{array}$$

And our task is to use the information in {*x*^*m*^, *y*^*m*^} to infer the function *f* : *x* → *y* that produces the best possible label field. 

### 3.2. The PP Model

**Figure 2:**
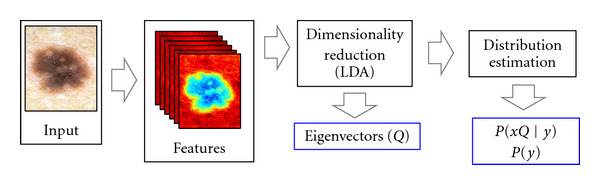
The training phase of our per-pixel (PP) model. Features are first computed, then the dimensionality of the featurespace is reduced using LDA. Posterior probabilities in this subspace are then estimated.

**Figure 3:**
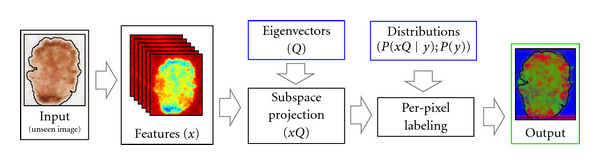
The testing phase of our per-pixel (PP) model. Features are computed as in the training phase. The projection *Q* is used to transform the features into the subspace determined in the training phase. Maximum a-posteriori estimation, using the posteriors estimated in the training phase, is then used to generate the label.

In this section, we briefly review our per-pixel (PP) estimation model [5]. An overview of the training and testing phases of the model is illustrated in Figures 2 and 3, respectively. Under this model, we assign the most probable label to each pixel independently

(2) $$y_{i}^{*}=\arg\;\underset{l_{j}}{\max}\left[P\left(y_{i}=l_{j}\;|\;x_{i}\right)\right];\;i=1,\ldots ,N_{P},$$

Applying Bayes' rule and simplifying, we arrive at the standard maximum likelihood formulation

(3) $$y_{i}^{*}=\arg\;\underset{l_{j}}{\max}\left[P\left(x_{i}\;|\;y_{i}=l_{j}\right)P\left(y_{i}\right)\right];\;i=1,\ldots ,N_{P}.$$

We model the posterior *P*(*x* | *y* = *l*) as a set of *N*_*L*_ multivariate normal distributions *P*(*x* | *y* = *l*_*j*_) = *N*(*μ*_*l*_*j*__, Σ_*l*_*j*__), whose parameters (*μ*_*l*_*j*__, Σ_*l*_*j*__) are estimated using the training set {*x*^*m*^, *y*^*m*^}. We model *P*(*y*) as a discrete distribution. Let *N*_*Yi*_ represent the number of elements in *y*^*m*^ that assume the value *l*_*i*_, then

(4) $$P\left(y_{i}\right)=\frac{N_{Yi}}{\Sigma_{j=1}^{N_{L}}N_{Yj}}.$$

We also normalize the probabilities across the label set, which are later used as features in the CRF model. The normalized likelihood that a pixel *i* is associated with the label *l*_*j*_ is

(5) $${ℒ}_{i,j}=\frac{P\left(x_{i}\;|\;y_{i}=l_{j}\right)}{\sum_{k=1}^{N_{L}}P\left(x_{i}\;|\;y_{i}=l_{k}\right)}.$$

In order to examine the model's performance across the entire sensitivity/specificity range, we consider many thresholds *T* on *ℒ*_*i*,*j*_ over the range [0,1] and label pixels accordingly.

As the number of channels (*N*_*C*_) in the images grows, we perform supervised dimensionality reduction on the observations *x* to focus the predictive power of our dataset onto a smaller subset of parameters. Linear discriminant analysis (LDA) is used to determine the subspace of *x* which best separates the labels [14].

LDA performs an eigenvalue decomposition of a scatter matrix representing the ratio of between-class covariance to within-class covariance. It returns a matrix of eigenvectors *Q* ∈ ℝ^*N*_*c*_×*N*_*L*_−1^ which projects observations (*x*) from *N*_*C*_ dimensions to *N*_*L*_ − 1

(6) $$\begin{array}{c} Q=\text{eig}\left(S_{w}^{-1}S_{b}\right),\\ S_{w}=\overset{N_{L}}{\underset{i=1}{\sum }}\Sigma_{l_{i}},\\ S_{b}=\overset{N_{L}}{\underset{i=1}{\sum }}\left(\mu_{l_{i}}-\mu \right){\left(\mu_{l_{i}}-\mu \right)}^{T}, \end{array}$$

where *μ* is the overall mean of *x* across all images and classes. Once the projection *Q* is determined, the posteriors are estimated, likelihoods are computed, and inference is performed in this subspace (*xQ*)

(7) $$\begin{array}{c} P\left(xQ\;|\;y=l_{j}\right)=N\left(\mu_{l_{j}}^{Q},\Sigma_{l_{j}}^{Q}\right);\;j=1,\ldots ,N_{L},\\ y_{i}^{*}=\arg\;\underset{l_{j}}{\max}\left[P\left(x_{i}Q\;|\;y_{i}=l_{j}\right)P\left(y_{i}\right)\right];\;i=1,\ldots ,N_{P}, \end{array}$$

(8) $${ℒ}_{i,j}=\frac{P\left(x_{i}Q\;|\;y_{i}=l_{j}\right)}{\sum_{k=1}^{N_{L}}P\left(x_{i}Q\;|\;y_{i}=l_{k}\right)},$$

where the superscript *Q* (*μ*_*l*_*j*__^*Q*^, Σ_*l*_*j*__^*Q*^) is used to differentiate the label means/covariances in this subspace from the original space in which the observations were performed (*μ*_*l*_*j*__, Σ_*l*_*j*__).

### 3.3. The CRF Model

**Figure 4:**
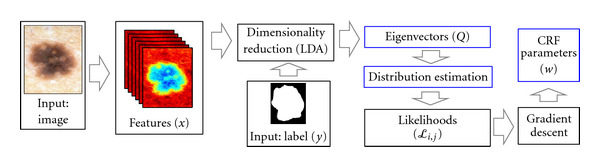
The training phase of our CRF model. We follow the same procedure as in our PP model up until the posteriors are estimated. We then calculate pixel likelihoods and use these as node features in our CRF model. We infer CRF parameters using gradient descent.

**Figure 5:**
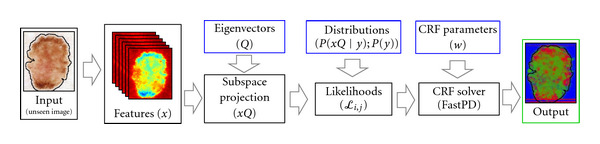
The testing phase of our CRF model. After the likelihoods are computed, we use the CRF parameters from the training phase, and the software FastPD to generate label fields.

In this section, we seek to improve upon the PP model developed in previous work [5] and described in Section 3.2. We present an overview of conditional random fields (CRFs) in Section 3.3.1. In Section 3.3.2, we describe how the CRF parameters can be determined empirically using maximum likelihood estimation (MLE) [15]. In Section 3.3.3, we discuss practical considerations for finding these parameters, including how to estimate the partition function [16] and how to regularize the likelihood expression [15]. In Section 3.3.4, we solve the MLE formulation via gradient-based methods. An overview of the training and testing phases of our CRF model is illustrated in Figures 4 and 5, respectively.

#### 3.3.1. Overview

The CRF model is an undirected graphical model that is naturally suited to represent and exploit the dependencies between observations, such as neighboring pixels in an image [15]. The probability that a label field *y* is associated with the image *x* under model parameters *w* is given by

(9) $$P\left(y\;|\;x;w\right)=\frac{1}{Z\left(x,w\right)}\exp\left(-E\left(y,x;w\right)\right),$$

where the function *Z*(*x*, *w*), known as the partition function, is used to normalize the probabilities for given values of *x* and *w*

(10) $$Z\left(x,w\right)=\underset{y}{\sum }\exp\left(-E\left(y,x;w\right)\right).$$

The energy function *E* represents the linear of combination of features employed by the model and is parameterized by the weight vector *w*

(11) $$E\left(y,x;w\right)=\overset{N_{W}}{\underset{k=1}{\sum }}w_{k}\Phi_{k}\left(y,x\right).$$

Given an undirected graph *𝒢* = (*𝒱*, *ℰ*), where *𝒱* represents the nodes (i.e., pixels) of an observation, *ℰ* represents the *dependencies* between nodes (throughout this document, *ℰ* is the 4-connected set of neighboring pixels), and the energy function *E* is the weighted sum of features Φ_*i*_(*y*, *x*). Features can either operate over the nodes of the graph (Φ^*V*^), or over its edges (Φ^*E*^)

(12) $$\begin{array}{c} \Phi^{V}\left(y,x\right)=\underset{i\in 𝒱}{\sum }\phi \left(y_{i},x_{i}\right),\\ \Phi^{E}\left(y,x\right)=\underset{\left(i,j\right)\in ℰ}{\sum }\phi \left(y_{i},y_{j},x_{i},x_{j}\right), \end{array}$$

In order for the model to be tractable, edge features Φ_*i*_^*E*^(*y*, *x*), and their corresponding weights must adhere to certain constraints. Let *𝔼* represent the set of edge features. The following constraints must be satisfied [17]

(13) $$\begin{array}{c} w_{i}>0\;\forall i\in 𝔼,\\ \phi^{E}\left(y_{i},y_{j},x_{i},x_{j}\right)=0\;\forall \left(y_{i},y_{j}\right)\;\text{s}.\text{t}.\;y_{i}=y_{j}. \end{array}$$

Strictly speaking, the second constraint can be replaced with the more general constraint that edge feature functions be *submodular* [18]. However, throughout this document, we will impose this stricter constraint which can be interpreted as “an edge cost is only incurred across nodes with differing labels.”

A CRF solver is one that, given observations *x* and parameters *w*, can find the most likely labeling y*

(14) $$y^{*}⟵\arg\;\underset{y}{\max}\;P\left(y\;|\;x;w\right).$$

We use the software FastPD [19, 20], which can exactly solve (14), under the constraints imposed above.

#### 3.3.2. Determining MLE Parameters

Since the emphasis of our work is on a general model capable of performing a variety of tasks, it is crucial that model parameters (*w*) be determined automatically from training data via empirical means. In this section, we derive the partial derivatives of the likelihood function which can be used by gradient-based methods to compute *w*.

Since the observations {*x*^*m*^, *y*^*m*^} are assumed to be independent. The likelihood of the data, given the set of parameters, is equal to the product of the probabilities in the observed set, under those parameters

(15) $$\ell \left(w\right)=\overset{N_{I}}{\underset{m=1}{\prod }}P\left(y^{m}\;|\;x^{m};w\right).$$

The maximum likelihood estimator is then

(16) $$w^{*}=\arg\;\underset{w}{\max}\overset{N_{I}}{\underset{m=1}{\prod }}P\left(y^{m}\;|\;x^{m};w\right).$$

If we can find the partial derivatives ∂*ℓ*/∂*w*_*i*_, we can optimize *w* using gradient-based methods. We begin by expressing the likelihood function *ℓ*(*w*) in terms of *w*

(17) $$\begin{array}{cc} w^{*} & =\arg\;\underset{w}{\max}\overset{N_{I}}{\underset{m=1}{\prod }}P\left(y^{m}\;|\;x^{m};w\right)\\ & =\arg\;\underset{w}{\max}\overset{N_{I}}{\underset{m=1}{\sum }}\ln\left(P\left(y^{m}\;|\;x^{m};w\right)\right)\\ & =\arg\;\underset{w}{\max}\overset{N_{I}}{\underset{m=1}{\sum }}\left(-E\left(y^{m},x^{m},w\right)-\ln\left(Z\left(x,w\right)\right)\right)\\ & =\arg\;\underset{w}{\min}\overset{N_{I}}{\underset{m=1}{\sum }}(\overset{N_{W}}{\underset{k=1}{\sum }}w_{k}\Phi_{k}\left(y^{m},x^{m}\right)\\ & \;\;\;\;\;\;\;+\ln\left[\underset{y}{\sum }\exp\left(-\overset{N_{W}}{\underset{k=1}{\sum }}w_{k}\Phi_{k}\left(y,x^{m}\right)\right)\right]). \end{array}$$

Solving for the partial derivatives, we get the following expression for the gradients of the likelihood function:

(18) $$\begin{array}{cc} \frac{\partial \ell }{\partial w_{i}} & =\overset{N_{I}}{\underset{m=1}{\sum }}(\Phi_{i}\left(y^{m},x^{m}\right)\\ & \;\;\;\;+\frac{\sum_{y}-\Phi_{i}\left(y,x^{m}\right)\exp\left(-\sum_{k=1}^{N_{w}}w_{k}\Phi_{k}\left(y,x^{m}\right)\right)}{\sum_{y}\exp\left(-\sum_{k=1}^{N_{w}}w_{k}\Phi_{k}\left(y,x^{m}\right)\right)}). \end{array}$$

However, we now come to an impasse. The second term of (18) would have us iterating over all possible label fields *y*. For a binary classification task over a modestly sized image of 256 × 128, this would require a summation over 2^256×128^ ≈ 2 × 10^9000^ labelings. Clearly this is intractable, and we must resort to estimating this second term.

#### 3.3.3. Practical Considerations

In order to derive CRF parameters with grid-structured models for even modestly sized images, a method to estimate the partition function is required. Inspired by [21], we employ one of the simplest estimation methods and approximate the partition function using saddle-point approximation (SPA) [16]

(19) $$\begin{array}{c} \underset{y}{\sum }\Phi \left(y,x\right)\approx \Phi \left(y^{*},x\right),\\ y^{*}⟵\arg\;\underset{y}{\max}\;P\left(y\;|\;x;w\right). \end{array}$$

We also introduce an additional practical consideration. Since gradient-based methods will be used to determine *w*, we regularize the likelihood function (*ℓ*(*w*)) by the squared L2 norm of the parameters [15] to penalize large weight vectors (since scalar multiples of a weight vector produce identical results). This makes the resulting likelihood function *strictly convex*. The regularized likelihood is then

(20) $$\begin{array}{cc} \ell \left(w\right) & =\overset{N_{I}}{\underset{m=1}{\sum }}\left(\overset{N_{W}}{\underset{k=1}{\sum }}w_{k}\Phi_{k}\left(y^{m},x^{m}\right)-\ln\underset{y}{\sum }\exp\overset{N_{W}}{\underset{k=1}{\sum }}w_{k}\Phi_{k}\left(y,x^{m}\right)\right).\\ & \;-\frac{{||w||}^{2}}{2\sigma^{2}} \end{array}$$

And the gradients become

(21) $$\begin{array}{cc} \frac{\partial \ell }{\partial w_{i}} & =\overset{N_{I}}{\underset{m=1}{\sum }}(\Phi_{i}\left(y^{m},x^{m}\right)\\ & \;\;+\frac{\sum_{y}-\Phi_{i}\left(y,x^{m}\right)\exp\left(-\sum_{k=1}^{N_{w}}w_{k}\Phi_{k}\left(y,x^{m}\right)\right)}{\sum_{y}\exp\left(-\sum_{k=1}^{N_{w}}w_{k}\Phi_{k}\left(y,x^{m}\right)\right)})-\frac{w_{i}}{\sigma^{2}}. \end{array}$$

Which under SPA becomes

(22) $$\frac{\partial \ell }{\partial w_{i}}\approx \overset{N_{I}}{\underset{m=1}{\sum }}\left(\Phi_{i}\left(y^{m},x^{m}\right)-\Phi_{i}\left(y^{*},x^{m}\right)\right)-\frac{w_{i}}{\sigma^{2}}.$$

#### 3.3.4. Implementation

We are now ready to implement a gradient-based method to estimate the CRF parameter vector *w*. Given an initial weight vector *w*^0^, the gradients of the likelihood function are estimated as per (22). These gradients are used to update the weight vector, which in turn is used to estimate a new set of gradients. This process is repeated until convergence.

We have observed (as does [21]) that gradient methods using saddle point approximation lead to oscillating behavior. Therefore, we keep a record of the best empirical set of parameters found, rather than the parameters of the final iteration. We also enforce the constraint from (13) that weights for edge-based features must remain positive.

In addition to the training set ({*x*, *y*}), the algorithm also requires an initial weight vector (*w*^0^), a regularization factor (*σ*^2^  ), a step size (*γ*), and termination conditions (convergence criteria: *ϵ*; maximum number of iterations: *N*_itr_). The algorithm has been found to be robust to these additional parameters. Pseudocode of our implementation is presented in Algorithm 1.

## 4. Results

Previous work has demonstrated our model's ability to generalize to many applications [5]. Here, we focus on a single application (lesion segmentation) and present results for our two models. We also compare our results to 5 previously published methods (KPP [6], JSEG [7], DTEA [8], SRM [9], and FSN [10]).

The dataset consists of 116 images from dermoscopy atlases [22, 23], which were acquired by a several dermatologists in separate practices using differing equipment. The images have not been properly color calibrated. Since the goal was to create a difficult dataset, 100 of the 116 lesions were selected to be particularly challenging to segmentation algorithms [7]. We intentionally chose a simplistic featureset to emphasize the power of the models under consideration.

The features employed were 5 Gaussian, and 5 Laplacian of Gaussian filters applied a various scales (*σ* = [1.25,2.5,5, 10,20]) in each channel of the image in CIE L*a*b* space. The responses of these filters represent the observations *x* (where *N*_*C*_ = 30). Each image was expertly segmented by a dermatologist. These ground truth labelings are denoted as *y*.

For all experiments, 10-fold cross-validation was employed. The dataset was randomly divided into 10 groups, and label fields for each group of images were determined using model parameters which were estimated from the observations in the 9 other groups. In both the PP and CRF models, all steps after the computation of features (refer to Figures 2 and 4) were included within the cross-validation loop including determining the projection *Q*, estimating the prior/posteriors, determining CRF parameter vector *w*, and so forth.

### 4.1. The PP Model

We begin by summarizing previous results on how our PP model faired on this dataset. A more detailed analysis, including the relative contribution of various aspects of the model (including features, dimensionality reduction, and classification method), can be found in our previous work [5].

**Figure 6:**
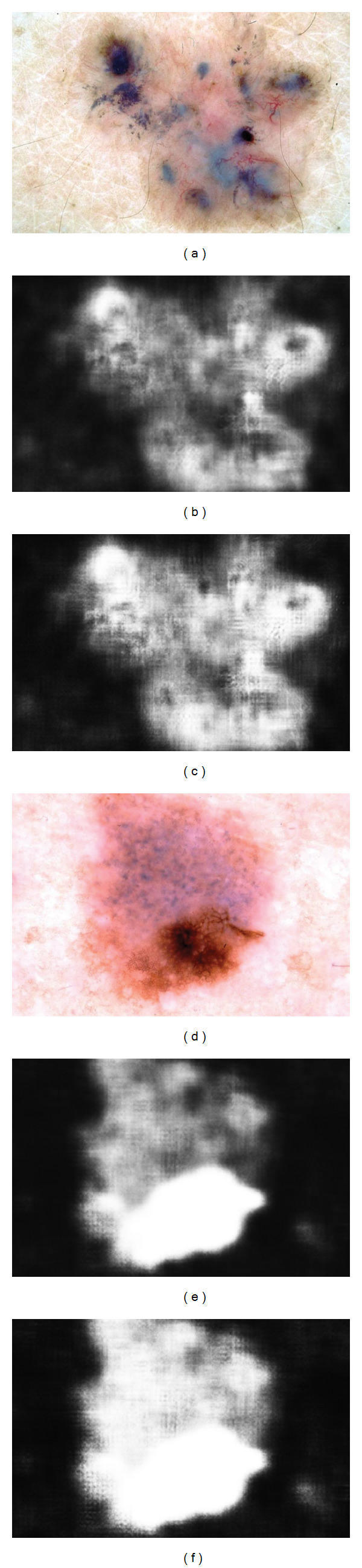
The effect of L* normalization on the segmentation likelihoods. left column: original dermoscopic image; middle: segmentation likelihoods (*ℒ*_*i*,lesion_) before L* normalization; right: after L* normalization.

**Figure 7:**
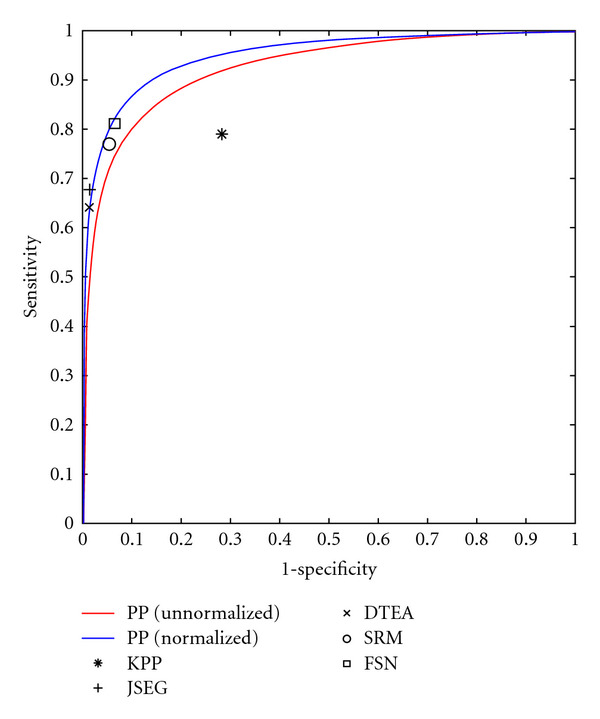
ROC curve comparing our PP model before normalization (red line) and after normalization (blue line) to 5 previously published methods.

Since that time, we have discovered that we can partially compensate for the lack of color calibration by subtracting the mean of the L* channel before computing features. While not as desirable as full color and lighting calibration [24], this procedure at least compensates for various camera exposure levels, as can be seen in the resulting PP likelihood maps in Figure 6 (as calculated by (8)).  Figure 7 illustrates a ROC curve comparing the performance of our PP model (before and after normalization) to the segmentation algorithms KPP [6], JSEG [7], DTEA [8], SRM [9], and FSN [10]. Our method performs comparably to JSEG, DTEA, SRM, and FSN and outperforms KPP although only KPP, DTEA, and FSN algorithms were able to generate results for all 116 images.  Table 1 summarizes the results. 

### 4.2. The CRF Model

As described in Section 3.3, the CRF model operates over an undirected graph *𝒢* = (*𝒱*, *ℰ*) and consists of node features (Φ^*V*^(*y*, *x*)) and edge features (Φ^*E*^(*y*, *x*)). The graph structure employed was the 4-connected set of neighboring pixels. Our featureset contains 2 features: one over the nodes and one over the edges. The node features are the likelihoods as computed by (8) of the PP model as in Section 4.1, and the edge features are set to the CIE L* intensity difference between neighboring pixels, if the labels of said pixels differ

(23) $$\begin{array}{cc} \Phi_{1}^{V}\left(y,x\right) & =\underset{i\in 𝒱}{\sum }\frac{P\left(x_{i}Q\;|\;y_{i}\right)}{\sum_{j=1}^{N_{L}}P\left(x_{i}Q\;|\;y_{i}=l_{j}\right)},\\ \Phi_{2}^{E}\left(y,x\right) & =\underset{\left(i,j\right)\in ℰ}{\sum }\left|L^{*}\left(x_{i}\right)-L^{*}\left(x_{j}\right)\right|1_{y_{i}\neq y_{j}}, \end{array}$$

where we use 1_*y*_*i*_≠*y*_*j*__ to denote the indicator function (i.e., 1_*y*_*i*_≠*y*_*j*__ evaluates to 1 if *y*_*i*_ ≠ *y*_*j*_; 0 otherwise)

While the method described in Section 3.3 is general enough to handle an arbitrary number of node and edge features, there are 2 reasons why we chose only one of each. To begin, we seek to make the comparison between the PP model and the CRF model as meaningful as possible. Using the likelihoods from the PP model as the node feature is an elegant way to evaluate the improvements realized by the CRF model. Note that with this choice of features, the CRF model with weight vector *w* = [1,0] gives identical results to the PP model. Additionally, the saddle-point method for approximating the partition function seems to degrade as the number of features increases. We note, however, that even in studies where the partition function can be computed exactly (because the CRF graph contains no loops), the loss incurred by such *piecewise training* methods is negligible [25].

**Figure 8:**
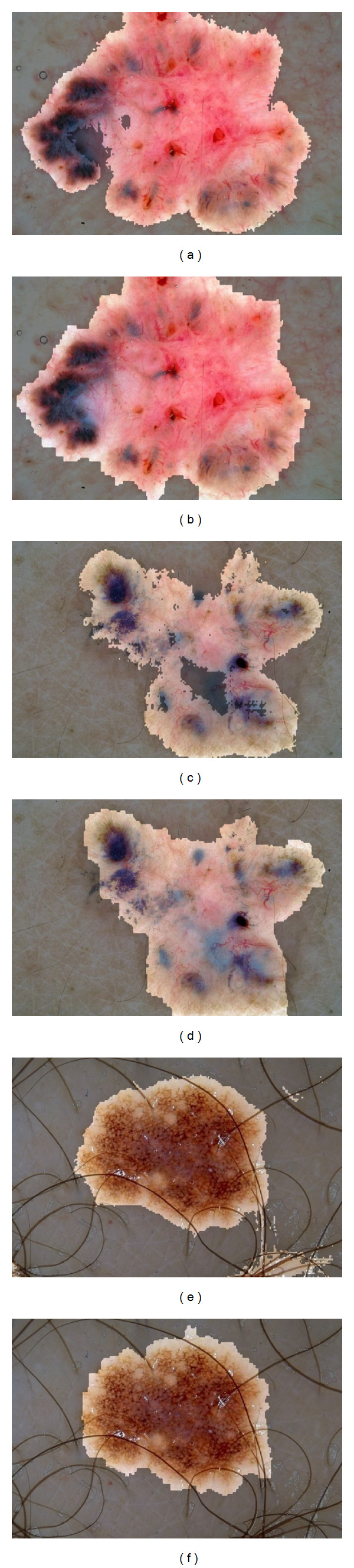
Comparing segmentations from our PP model (left) and CRF model (right). Since the CRF model relaxes the assumption of pixel independence in the PP model, it is able to smooth over local discontinuities. The result is better segmentations which fill in “holes” and remove “noise.”

Figure 8 compares some segmentations produced by the PP and CRF model. By relaxing the assumption of independence in the PP model, the CRF model is able to smooth over small areas of discontinuity, filling in “gaps” in segmentations, and removing noise. In Figures 8(a) and 8(c), the “holes” in the resulting PP segmentations do not manifest in the CRF segmentations (Figures 8(b) and 8(d)) due to the model's holistic search for the best label *field*, rather than best individual label. Additionally, while the PP model is already fairly robust to occluding hair (Figure 8(e)), the CRF model is even more robust, able to smooth over misclassifications due to artifacts.

We also tested the stability of the CRF model with respect to regularization and the hyperparameter *σ*^2^. Varying *σ*^2^ (to assume values in the range [10^−6^, Inf⁡]) had little effect on performance of the model on this particular dataset. In spite of the seemingly ineffectual nature of this parameter, we do not remove it from the model since the emphasis of this work is on *general* models for ASLD. The effect of *σ*^2^ in general (over many tasks in ASLD) has yet to be determined.

**Figure 9:**
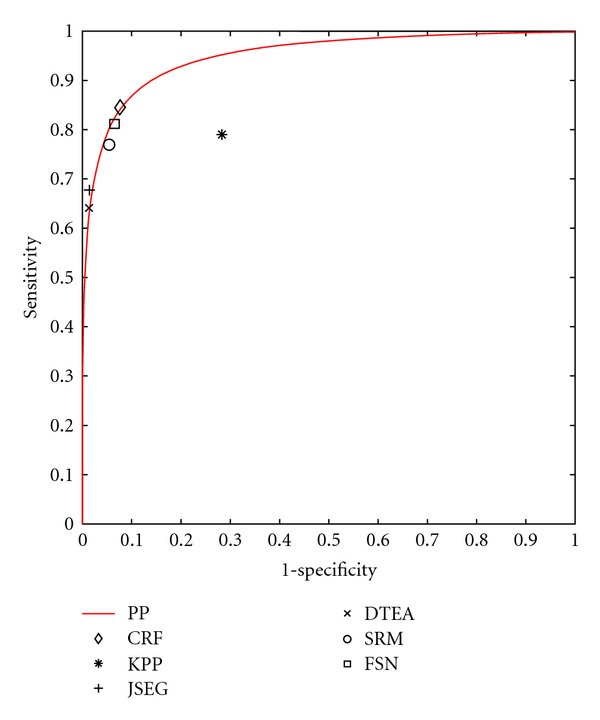
ROC curve comparing our CRF model (diamond) to our PP model (line) and 5 previously published methods.

While subjectively, the CRF model offers substantial improvements; objectively, the CRF model is a marginal improvement over the PP model.  Figure 9 shows an ROC curve comparing the CRF's performance to that of the PP model and previously published methods, and Table 1 summarizes the results. 

## 5. Conclusions

In this paper, we have generalized several common problems in ASLD into a single formulation. We also presented 2 probabilistic models capable of solving the formulation, and described how supervised learning can be used to determine all model parameters. Since the parameters for the resulting models can all be determined automatically from training data, it is hoped that these models can be applied quickly and effectively to a variety of relevant tasks in ASLD.

While both methods perform comparably to previously published methods, the qualitative improvements realized by CRF model aren't reflected in the quantitative score. Unlike the PP model, the CRF model does not assign labels to pixels independently. Rather, the CRF model selects the best label *field* to assign to an image. This allows the CRF model to fill in “holes” and smooth out noise that would otherwise appear.

The discrepancy between the objective and subjective performance of the CRF model implies that our evaluation metric (pixel-wise sensitivity and specificity) may be less than ideal. Therefore, future work will explore the use of alternate evaluation metrics [26, 27].

Even though the models presented are competitive, there are many potential directions in which they can be improved upon even further. In our grid-structured CRF model, we must resort to approximating the partition function due to the computational complexity of calculating it exactly. Imposing a tree-based structure over the image [25] would enable the exact computation of the partition function via dynamic programming and should lead to more reliable CRF parameters. Replacing our gradient-based method for determining CRF parameters with a max-margin formulation [13] is another possible way to increase the reliability of the resulting parameters. We can also induce non-linearities into the model by replacing the linear dimensionality reduction step (LDA) with its nonlinear counterparts (i.e., KLDA [28]). Finally, the use of semi-supervised learning techniques may be used to decrease the cost of acquiring expertly annotated datasets [29].

## Figures and Tables

**Algorithm 1:**
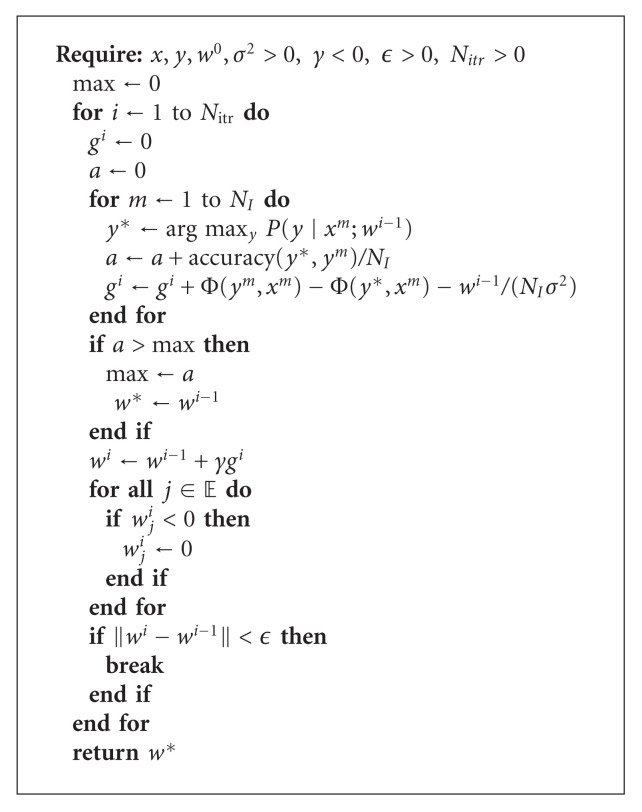
Calculating the CRF parameter vector *w* using gradient descent and saddle-point approximation.

**Table 1:** Comparison of our PP model's ability to segment lesions to our CRF model and 5 previously published methods.

		Performance			
		Method		PP (nearest pt.)	
Method	*n*	Sens	Spec	Sens	Spec
CRF	116	0.845	0.924	0.843	0.921
KPP [6]	116	0.765	0.770	0.941	0.763
JSEG [7]	91	0.627	0.987	0.677	0.980
DTEA [8]	116	0.597	0.986	0.638	0.985
SRM [9]	112	0.790	0.946	0.773	0.957
FSN [10]	116	0.808	0.934	0.814	0.939
